# Usage of and satisfaction with Integrated Community Case Management care in western Uganda: a cross-sectional survey

**DOI:** 10.1186/s12936-021-03601-9

**Published:** 2021-01-30

**Authors:** James S. Miller, Palka Patel, Sara Mian-McCarthy, Andrew Christopher Wesuta, Michael Matte, Moses Ntaro, Shem Bwambale, Jessica Kenney, Geren S. Stone, Edgar Mugema Mulogo

**Affiliations:** 1 Massachusetts General Hospital, Boston, MA USA; 2 Harvard Medical School, Boston, MA USA; 3 Global Health Collaborative MUST Uganda, Mbarara, Uganda; 4 Bugoye Community Health Collaboration, Bugoye, Uganda; 5 Indiana University School of Medicine, Indianapolis, IN USA; 6 UNICEF USA, New York, NY USA; 7 Mbarara University of Science and Technology, Mbarara, Uganda; 8 Bugoye Health Centre, Bugoye, Uganda

**Keywords:** Village health workers, Community health workers, Integrated Community Case Management, Patient satisfaction, Community perspective

## Abstract

**Background:**

In some areas of Uganda, village health workers (VHW) deliver Integrated Community Case Management (iCCM) care, providing initial assessment of children under 5 years of age as well as protocol-based treatment of malaria, pneumonia, and diarrhoea for eligible patients. Little is known about community perspectives on or satisfaction with iCCM care. This study examines usage of and satisfaction with iCCM care as well as potential associations between these outcomes and time required to travel to the household’s preferred health facility.

**Methods:**

A cross-sectional household survey was administered in a rural subcounty in western Uganda during December 2016, using a stratified random sampling approach in villages where iCCM care was available. Households were eligible if the household contained one or more children under 5 years of age.

**Results:**

A total of 271 households across 8 villages were included in the final sample. Of these, 39% reported that it took over an hour to reach their preferred health facility, and 73% reported walking to the health facility; 92% stated they had seen a VHW for iCCM care in the past, and 55% had seen a VHW in the month prior to the survey. Of respondents whose households had sought iCCM care, 60% rated their overall experience as “very good” or “excellent,” 97% stated they would seek iCCM care in the future, and 92% stated they were “confident” or “very confident” in the VHW’s overall abilities. Longer travel time to the household’s preferred health facility did not appear to be associated with higher propensity to seek iCCM care or higher overall satisfaction with iCCM care.

**Conclusions:**

In this setting, community usage of and satisfaction with iCCM care for malaria, pneumonia, and diarrhoea appears high overall. Ease of access to facility-based care did not appear to impact the choice to access iCCM care or satisfaction with iCCM care.

## Background

Village health workers (VHW) in Uganda provide a range of health promotion and health education services. In some areas, VHWs deliver Integrated Community Case Management (iCCM) care, providing initial assessment of children under 5 years of age as well as protocol-based treatment of malaria, pneumonia, and diarrhoea for eligible patients. Little is known about community satisfaction with or perspectives on iCCM care.

In 2014, an iCCM Child Health and Nutrition Research Initiative Advisory Group, in collaboration with the Community Case Management Operational Research Group, systematically identified “global research gaps and resource priorities for [iCCM]” [1]. Their top-ranked research objective was to “assess perceptions of beneficiaries and levels of community satisfaction in community health workers’ capacity.” Since then, one other study, also in Uganda, has assessed community usage and perspectives on iCCM services, reporting that 53% of those who had received iCCM services were satisfied with care [2]. Other studies have examined satisfaction with home-based fever or pneumonia management alone rather than iCCM; one study in Ghana demonstrates that a majority of caregivers rated home-based management of fever by a VHW as “good” or “excellent” [3], while another study in Kenya describes caregivers’ expressed preference for home-based pneumonia care over facility-based care [4]. By comparison, a study in Pakistan of home-based pneumonia and diarrhoea care described very low use of and confidence in community health workers to provide care for these conditions [5].

This study examines usage of and satisfaction with iCCM care overall, as well as respondents’ confidence in VHWs’ ability to manage each of the three conditions (fever/malaria, pneumonia, and diarrhoea). The analytic component of the study further assesses the association between time required to travel to the household’s preferred health facility and usage of and satisfaction with iCCM care.

## Methods

### Study setting

Bugoye is a rural sub-county of Kasese District in western Uganda, in the foothills of the Rwenzori Mountains. Its mountainous geography limits access to facility-based care for households in more remote villages. The public-sector health system in Bugoye consists of a larger health centre, several smaller satellite facilities, and VHWs in each village. The VHWs in Bugoye are chosen by their communities and serve as unpaid volunteers, in keeping with national standards; they do receive a transportation stipend and other nonmonetary incentives (e.g., raincoats or flashlights) when they attend trainings at a local health centre.

### Survey participants and methods

The data presented here come from a larger cross-sectional household survey conducted in December 2016 examining interaction with VHWs, healthcare usage patterns, use of iCCM care, satisfaction with care, health-related behaviours, and demographic information for households with young children. A new survey tool was developed for this study as no existing tool covering these concepts was available (see Additional file 1). The survey instrument was translated from English to Lukonjo (the local language), with the translation reviewed and edited by multiple staff members fluent in English and Lukonjo.

The survey was conducted in 8 villages in Bugoye sub-county in which VHWs were providing iCCM services at that time. Households were eligible for the survey if they resided in one of the included villages and contained one or more children under 5 years of age. This report examines a subset of the survey questions focusing on caregivers’ experiences of iCCM care. Other survey results will be reported separately.

Prior to the survey, VHWs in each village created a sampling frame of households containing children under 5 years old. After data collection commenced, it became clear that the sampling frames for some villages were significantly inaccurate, so the survey collection team corrected the sampling frame and modified the stratified sampling accordingly. The corrected sampling frame contained 909 households. The revised sample size (using the corrected sampling frame) was calculated based on the binary outcome of a household having visited a VHW in the month prior to the survey, with this proportion assumed to be 50%, with a 5% margin of error. A final sample size of 271 households was calculated using the Select Statistical Services Population Proportion Sample Size Calculator [6]. A finite population correction was used given the relatively small number of eligible households. A random sample of households was selected, stratified by village and weighted by the number of eligible households in each village. Study staff visited each selected household in December 2016 and confirmed eligibility (i.e., that the household contained a child under 5) and that a respondent over 18 years of age who spoke English or Lukonjo (the local language) was available. If no eligible respondent was available, study staff made a second trip to the household. If a household was ineligible, declined to participate, or no respondent was available at either visit, a replacement household was randomly selected from the sampling frame for that village.

The initial inaccurate sampling frame resulted in inaccurate stratified sampling calculations as well (since some villages had substantially more eligible households identified in the initial sampling frame compared with the corrected sampling frame). Because of this issue, excess surveys were conducted in several villages before the issue was identified and the sampling frame corrected. In this situation, surveys were randomly dropped from the sample for that village until the correct number of surveys for the revised stratified sample was achieved.

Survey questions assessing respondents’ satisfaction with iCCM care and confidence in VHWs’ abilities used 5-point Likert scales. Travel time to the health facility was captured as a categorical variable (less than 30 min, 30–59 min, 1–2 h, 2–3 h, 3–4 h, more than 4 h); due to the small number of respondents in the higher categories, a combined category of more than 2 h was used for the regression models described below.

All respondents provided written consent prior to participation. Study staff recorded survey responses using the Research Electronic Data Capture (REDCap) application on tablet computers [7]. Ethical approval was granted by the Partners Healthcare Institutional Review Board and Research Ethics Committee at the Mbarara University of Science and Technology.

### Data analysis

Data were cleaned in REDCap and analysed in Stata Version 15 (StataCorp, College Station, TX). Logistic regression models, accounting for stratified survey design, were used to assess the relationship between outcomes of interest (usage of and overall satisfaction with iCCM care) and travel time required to access facility-based care. For overall satisfaction, ordered logistic regression was used, with the outcome variable maintained as a 5-point Likert scale and travel time modeled as a categorical variable (as described above). Usage of iCCM care in the last month represents a binary outcome variable, with travel time again modeled as a categorical variable.

## Results

**Fig. 1:**
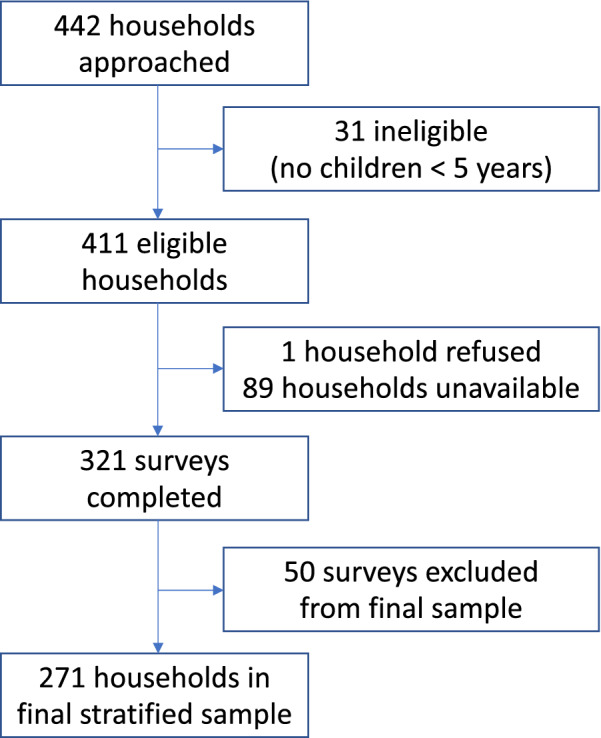
Study flow diagram

Study staff approached a total of 442 households over a 2-week period. Of these, 31 were determined to be ineligible as they did not contain a child under 5 years. Of the 411 eligible households, 1 declined to participate and 89 had no appropriate respondent available at either visit by study staff. Due to the discrepancy between the initial sampling frame that was used to allocate households among research assistants and the corrected sampling frame, 50 additional surveys were completed but were not included in the final sample (to allow for an appropriately stratified sample based on the final sampling frame). Thus, a total of 321 surveys were completed yielding a response rate of 78%, with 271 surveys included in the final stratified sample (Fig. 1).

Respondents were predominantly female (61%), with a mean age of 35 years; 53% had completed primary school. Households heads were predominantly male (71%), with mean age of 41 years; 64% had completed primary school. When asked about their experience traveling to their preferred health facility, 24% of respondents reported travel time less than 30 min, 37% reported 30–59 min, 30% reported 1–2 h, and 9% reported over 2 h; 73% reported walking to the health facility, while 18% traveled by hired motorcycle or car, 8% by private motorcycle or car, and 1% by bicycle (Table 1).

**Table 1:** Respondent and household demographic information

Measure	n (%) or mean (range)
Female respondent	163 (61%)
Respondent age in years	35 (18–93)
Respondent completed primary school	138 (53%)
Male household head	192 (71%)
Household head age in years	41 (20–89)
Household head completed primary school	168 (64%)
Time spent travelling to preferred health facility	
Less than 30 min	66 (24%)
30–59 min	99 (37%)
1–2 h	81 (30%)
2–3 h	20 (7%)
3–4 h	3 (1%)
More than 4 h	2 (1%)
Means of travel to preferred health facility	
Walking	199 (73%)
Hired motorcycle or car	49 (18%)
Private motorcycle or car	21 (8%)
Bicycle	2 (1%)
Village	
Bugoye	36 (13%)
Ihani	30 (11%)
Kanyaminigo	45 (17%)
Kikokera	21 (8%)
Muramba 1	37 (14%)
Ndugutu East	31 (11%)
Rwakingi 1B	27 (10%)
Ruboni	44 (16%)

Of those surveyed, 92% stated that a child in their household had seen a VHW for iCCM care at some point (iCCM care became available 3 years prior to the survey), with 55% reporting a child receiving iCCM care in the month prior to the survey. Of those who had taken a child for iCCM care, 87% stated they contact the VHW by going to the VHW’s house, and 97% stated that the VHW was available promptly, though 14% could recall a time when they could not find the VHW. Results were similar for the subset of respondents with a child in their household who had received iCCM care in the month prior to the survey (Table 2).

**Table 2:** Usage of and satisfaction with iCCM services (reported for all respondents and for the subset of respondents with a child in their household receiving iCCM care in the last month)

Measure	All respondents n (%)	iCCM care in last month n (%)
Usage of iCCM services		
Ever seen VHW for iCCM care	249 (92%)	–
VHW available promptly*	241 (97%)	134 (99%)
Ever unable to find VHW when child was sick	39 (14%)	14 (10%)
Contact VHW most of the time or every time child is sick	158 (58%)	94 (69%)
When child is sick, contact VHW by going to her/his house	235 (87%)	120 (88%)
Seen VHW for iCCM care in last month*	136 (55%)	–
Satisfaction with iCCM services		
Overall experience of most recent time receiving iCCM care—rated very good or excellent*	150 (60%)	86 (63%)
Would have child seen by VHW in the future when child sick	263 (97%)	136 (100%)
Confidence in VHW’s overall abilities—rated confident or very confident	250 (92%)	133 (98%)
Confidence in VHW’s ability to manage fever—rated confident or very confident	249 (92%)	134 (99%)
Confidence in VHW’s ability to manage cough—rated confident or very confident	246 (91%)	127 (93%)
Confidence in VHW’s ability to manage diarrhoea—rated confident or very confident	249 (92%)	131 (96%)

iCCM: Integrated Community Case Management; VHW: village health worker

*Question only asked of respondents with a child in their household who had received iCCM care at some point

Of respondents who had seen a VHW for iCCM care, 60% rated their overall experience as “very good” or “excellent,” while 97% of respondents stated they would seek iCCM care in the future when a child becomes sick. Respondents expressed a high degree of confidence in their VHW, with 92% stating they were “confident” or “very confident” in the VHW’s overall abilities, with similar levels of confidence in VHWs’ abilities to manage fever, cough, and diarrhoea. Again, the subset of respondents with a child their household who had received iCCM care in the prior month expressed similar satisfaction with care and confidence in VHWs (Table 2).

Univariate logistic regression models did not demonstrate any notable relationship between travel time to the household’s preferred health facility and overall satisfaction with iCCM care or propensity to seek iCCM care. Similarly, for the subset of respondents with a child in their household who had received iCCM care in the month prior to the survey, there did not appear to be a relationship between travel time and overall satisfaction with iCCM care (Table 3).

**Table 3:** Logistic regression results

Measure	Odds ratio	95% CI	*p* value
Model 1: Univariate ordered logistic regression for the relationship between overall rating of iCCM care and travel time to the health facility			
Less than 30 min	(Reference category)		
30–59 min	0.90	0.47–1.72	0.74
1–2 h	0.79	0.41–1.52	0.47
More than 2 h	1.02	0.38–2.73	0.96
Model 2: Univariate ordered logistic regression for relationship between overall rating of iCCM care and travel time to the health facility, for the subset of respondents with a child in their household receiving iCCM care in the last month			
Less than 30 min	(Reference category)		
30–59 min	0.69	0.29–1.61	0.39
1–2 h	0.48	0.19–1.22	0.12
More than 2 h	0.75	0.24–2.33	0.61
Model 3: Univariate logistic regression for the relationship between a child in the household receiving iCCM care in the last month and travel time to the health facility			
Less than 30 min	(Reference category)		
30–59 min	1.26	0.65–2.46	0.49
1–2 h	1.17	0.59–2.33	0.65
More than 2 h	1.61	0.60–4.31	0.34

CI: confidence interval; iCCM: Integrated Community Case Management

## Discussion

This study demonstrates high levels of reported utilization of iCCM care, with 55% of respondents reporting use of iCCM care for a child in their household within the last month. Overall, respondents reported that VHWs were available to provide iCCM care when needed, as well as high levels of satisfaction with and confidence in iCCM care, both overall and for the specific domains of iCCM (malaria, pneumonia, and diarrhoea). It is curious that only 60% of respondents rated their most recent episode of iCCM care as “very good” or “excellent”, but 97% planned to seek iCCM care in the future, and over 90% expressed confidence in VHWs’ abilities across all domains. This discrepancy could suggest that respondents were only somewhat satisfied with iCCM care but still preferred it to facility-based care, or may reflect an issue with item validity or social desirability bias.

Access to iCCM care may particularly benefit households in more remote areas with less easy access a health facility, such as parts of Bugoye that are not connected to the road system due to its mountainous geography. However, logistic regression modelling did not demonstrate evidence of a relationship between travel time to households’ preferred health facility and usage of or satisfaction with iCCM care. This result could be due to broad community preference for iCCM care over facility-based care as suggested by a prior study of home-based pneumonia care in Kenya [4].

This study has at least five limitations. First, reported iCCM care usage could not be verified with clinical records, so estimates of iCCM care use overall and within the past month are limited by respondent recall. Second, respondents might have perceived research assistants as affiliated with the local health centre or iCCM programme, so responses might have been affected by social desirability bias or simply politeness toward their neighbors who serve as VHWs. Third, these data come from a larger survey with its sample size calculated based on a binary outcome, so the study was not designed or powered specifically for the use of a categorical outcome variable in the ordered logistic regression models described above. Fourth, while the survey instrument was carefully translated and reviewed by multiple staff members fluent in English and Lukonjo, the process of translation could nonetheless have affected item validity. Fifth, violence that occurred in this area of Uganda shortly prior to survey administration in 2016 may have decreased the response rate, as residents may been temporarily displaced or less willing to interact with the research assistants conducting the survey.

## Conclusions

This study suggests that usage of and satisfaction with iCCM care for malaria, pneumonia, and diarrhoea is high overall in this rural Ugandan setting. Ease of access to facility-based care did not appear to impact the choice to access iCCM care or satisfaction with iCCM care in this setting. Future research may elucidate other determinants of usage of and satisfaction with iCCM care. Variation in prior research findings in this field may be related in part to differences in culture, setting, and availability or cost of other care options. Allowing for differences in measurement approach, these results are broadly comparable to prior research in Uganda and Kenya, and are likely generalizable to other rural settings in Uganda and potentially other rural settings in sub-Saharan Africa.

## Supplementary information

**Additional file 1.** Survey instrument for the overall cross-sectional household survey.

## Data Availability

The dataset used during the current study is available from the corresponding author on reasonable request.

## Abbreviations

iCCM: Integrated Community Case Management
REDCap: Research Electronic Data Capture
VHW: Village health worker
