# Supplementary material for: Usage of and satisfaction with Integrated Community Case Management care in western Uganda: a cross-sectional survey
Source: Malar J. 2021 Jan 30;20:65. doi: 10.1186/s12936-021-03601-9 (PMC7847039; doi:10.1186/s12936-021-03601-9)
Supplement: Supplementary file 1 — Additional file 1. Survey instrument for the overall cross-sectional household survey. [file 12936_2021_3601_MOESM1_ESM.pdf]

2016 BIMI household survey

Interviewers: Please circle only 1 answer per question unless otherwise specified. Please choose "No response" if respondent unsure, unwilling, or unable to answer the question. Each question is to be read the participant as written.

A. SURVEY INFORMATION

101

Interviewer	Data entry 1	Data entry 2	Data review
Name:	Name:	Name:	Name:
ID:	ID:	ID:	ID:
Date:	Date:	Date:	Date:
Time:	Time:	Time:	Time:

102 Village:
- a. Bugoye
- b. Ihani
- c. Kanyaminigo
- d. Kikokera
- e. Muramba 1
- f. Ndugutu East
- g. Rwakingi 1B
- h. Ruboni

103 House Number

104 Head of household name: ____

105 Visit to household
- a. First attempt
- b. Second attempt

106 Consent
- a. Obtained
- b. Refused (STOP, GO TO NEXT HOUSEHOLD)
- c. No respondent at household (STOP, GO TO NEXT HOUSEHOLD)

B. HOUSEHOLD INFORMATION

READ: We will start with some basic questions about you and the members of your household.

201 What language would you prefer for the survey:
   a. Lukonjo
   b. English

202 Are you the head of the household:
   a. Yes
   b. No (GO TO QUESTION 207)

203 Respondent age: ___________ years (Use "999" if does not desire to share age)

204 Respondent gender
   a. Male
   b. Female

205 Respondent occupation (circle only primary occupation)
   a. Farmer (ie. "peasant farmer")
   b. Business
   c. Teacher
   d. Medical
   e. Driver
   f. Government worker
   g. Other (please specify): ____
   h. No response

206 Respondent highest education completed:
   a. No formal education/less than P1
   b. Some primary school (P1-P7)
   c. All primary school (completed P7)
   d. Some secondary school (S1-S4)
   e. Completed ordinary secondary school (completed S4)
   f. Some advanced secondary school (S4-S6)
   g. All advanced secondary school (completed S6)
   h. Some tertiary school
   i. Tertiary certificate, diploma, or degree
   j. No response

(GO TO QUESTION 215)

207 Head of household age: ___________ years

208 Head of household gender
   a. Male
   b. Female

209 Head of household occupation (circle only primary occupation)
   a. Farmer
   b. Business
   c. Teacher/Education
   d. Medical
   e. Driver
   f. Government
   g. Other (please specify: ____
   h. No response

210 Head of household highest education completed :
   a. No formal education/less than P1
   b. Some primary school (P1-P7)
   c. All primary school (completed P7)
   d. Some secondary school (S1-S4)
   e. Completed ordinary secondary school (completed S4)
   f. Some advanced secondary school (S4-S6)
   g. All advanced secondary school (completed S6)
   h. Some tertiary school
   i. Tertiary certificate, diploma, or degree
   j. No response

211 Respondent's relationship to head of household:
   a. Spouse
   b. Parent
   c. Grandparent
   d. Child
   e. Grandchild
   f. Sibling
   g. Friend
   h. Other family
   i. Other (please specify):____
   j. No response

212 Respondent's age: ___________ years

213 Respondent gender
   a. Male
   b. Female

214 Respondent highest education completed:

a. No formal education/less than P1
b. Some primary school (P1-P7)
c. All primary school (completed P7)
d. Some secondary school (S1-S4)
e. Completed ordinary secondary school (completed S4)
f. Some advanced secondary school (S4-S6)
g. All advanced secondary school (completed S6)
h. Some University
i. University Degree
j. No response

215 How many people, including yourself, live in your household? Please include all children and infants. Please do not include guests who do not live here regularly.

____

216 How many children under the age of 5 years live in your household? Please include all children and infants. Please do not include guests who do not live here regularly.

____

217 On average, what is the total monthly income of the members of this household?

a. 100,000 or less UGX
b. 100,001-200,000 UGX
c. 200,001-300,000 UGX
d. 300,001-400,000 UGX
e. 400,001-500,000 UGX
f. 500,001-600,000 UGX
g. 600,001-700,000 UGX
h. 700,001-800,000 UGX
i. 800,001-900,000 UGX
j. 900,001-1,000,000 UGX
k. More than 1,000,000 UGX

218 Do you have a mobile phone?

a. Yes
b. No
c. No response

219 Does your household have electricity?

a. Yes
b. No
c. No response

220 What is the main source of drinking water for members of your household?

a. Piped water to dwelling or plot
b. Public tap or standpipe
c. Open well or spring in yard or plot
d. Open public well or spring
e. Protected well or spring in yard or plot
f. Protected public well or spring
g. Borehole in yard or plot
h. Public borehole
i. Surface water (river/lake/dam)
j. Rain water
k. Tanker truck
l. Vendor
m. Bottled water
n. Other (specify) ________________________
o. No response

221 How many minutes does it take to get to the main source of drinking water, retrieve the water, and return home?

a. 0-10 mins
b. 11-20 mins
c. 21-30 mins
d. 31-45 mins
e. 46-60 mins
f. More than 60 mins
g. No response

222 What type of container do you primarily use to store water in?

a. Jerry can
b. Bucket with lid
c. Bucket without lid
d. Pots or saucepan
e. Other (specify): ____
f. No response

223 What kind of toilet facility do members of your household usually use?

a. Flush toilet
b. Vip latrine
c. Covered pit latrine no concrete slab
d. Covered pit latrine with concrete slab
e. Uncovered pit latrine no concrete slab
f. Uncovered pit latrine with concrete slab
g. Composting toilet or EcoSan
h. Bush **(**GO TO QUESTION 226)
i. Other (specify) ________________________________
j. No response

224 Do you share this toilet facility with other households?
   a. Yes
   b. No **(**GO TO QUESTION 226)
   c. No response **(**GO TO QUESTION 226)

225 How many households use this toilet facility? (write 99 if does not know) _________________

226 In a typical week, on how many days do you eat meat?
   a. 0 days
   b. 1-2 days
   c. 3-5 days
   d. 6-7 days
   e. No response

227 How often in the past 6 months did you have problems satisfying the food needs of the household?
   a. Never
   b. Seldom
   c. Sometimes
   d. Often
   e. Always
   f. No response

C. *HEALTH AND HEALTHCARE INFORMATION*

READ: Now I would like to talk about the health care your family receives.

301 To the nearest kilometer, approximately how far do you think it is to the nearest health center?
  a. Less than 1 km
  b. 1 km
  c. 2 km
  d. 3 km
  e. 4 km
  f. 5 or more km
  g. No response

302 What is the name of that health facility?
  a. Bugoye Health Center III
  b. Ibanda Health Center II
  c. Kilembe Mines Hospital
  d. Rwenzori mountaineering services (RMS)
  e. Bishop Masereka Medical Centre
  f. St. Paul Health Centre
  g. Kasese Town Council Clinic
  h. Manasi's Clinic
  i. Afiya Camiri Clinic
  j. Kasese Moslem Health III
  k. Alleluya Medical Centre
  l. Kasese Medical Centre
  m. Other: ____________________________________
  n. No response

303 Is this health facility, the primary site you would go to if you or a family member are in need of medical care?
  a. Yes (GO TO QUESTION 305)
  b. No

304 To which health facility would you go if you or a family member were in need of medical care?
  a. Bugoye Health Center III
  b. Ibanda Health Center II
  c. Kilembe Mines Hospital
  d. Rwenzori mountaineering services (RMS)
  e. Bishop Masereka Medical Centre
  f. St. Paul Health Centre
  g. Kasese Town Council Clinic
  h. Manasi's Clinic
  i. Afiya Camiri Clinic
  j. Kasese Moslem Health III
  k. Alleluya Medical Centre
  l. Kasese Medical Centre
  m. Other: ____________________________________

n. No response

305 What is your primary means of transport to the health facility?
a. Private car or motorcycle
b. Hired care or motorcycle
c. Animal or animal cart
d. Walking
e. Bicycle
f. Other (specify):____
g. No response

306 Do you spend any money on transport to the health facility?
a. Yes
b. No (GO TO QUESTION 308)
c. No response (GO TO QUESTION 308)

307 If you spend money on transport to get to the health facility, how much do you usually spend for a one-way trip?
a. 0-2000 UGX
b. 2001-5000 UGX
c. 5001-10,000 UGX
d. 10,001-20,000 UGX
e. More than 20,000 UGX
f. No response

308 How much time do you typically spend getting to that health facility for a 1-way trip? Please include waiting time for transport.
a. Less than half hour
b. 30-59 minutes
c. 1-1 hour 59 mins
d. 2-2 hours 59 mins
e. 3-3 hours 59 mins
f. 4 or more hours
g. No response

309 In the past year, has there been a time when a child in your household was sick, and you thought the child needed treatment, but you were not able to seek treatment at a health facility?
a. Yes
b. No (GO TO QUESTION 311)
c. No response (GO TO QUESTION 311)

310 In that situation, what was the main reason that you could not go to the health facility for treatment for your child?
a. Lack of money
b. Lack of time
c. Lack of trust in provider
d. Lack of transport

e. Lack of approval by spouse
f. Health center without medications
g. Other (specify):____
h. No response

READ: Now I would like to ask you a few more questions about all of the children under 5 years of age who lived in your household over the past year.

311 Have any children under 5 years of age who lived in your household died in the past year?
a. Yes
b. No **(**GO TO QUESTION 314)
c. No response **(**GO TO QUESTION 314)

312 How many children under 5 years of age died in the last year (12 months)? ____________

313 Fill out for each child who died <5 years old.

a. Child #1
b. How old was **Child #1:**
1. <1 month
2. ≥1 month – 11 months
3. 1 year old
4. 2 years old
5. 3 years old
6. 4 years old

c. What do you believe **Child 1** died from:
1. Malaria
2. Pneumonia
3. Diarrhea
4. Unknown
5. Other (specify):____

d. Child #2
e. How old was **Child #2:**
1. <1 month
2. ≥1 month – 11 months
3. 1 year old
4. 2 years old
5. 3 years old
6. 4 years old

f. What do you believe **Child 2** died from:
1. Malaria
2. Pneumonia
3. Diarrhea
4. Unknown
5. Other (specify):____

g. Child #3
h. How old was **Child #3:**
1. <1 month
2. ≥1 month – 11 months

3. 1 year old
4. 2 years old
5. 3 years old
6. 4 years old

i. What do you believe **Child 3** died from:
1. Malaria
2. Pneumonia
3. Diarrhea
4. Unknown
5. Other (specify):_______________________________________

READ: Now I would like to ask more about your experience of getting healthcare for your children.

314 When one of your children under 5 years old has been sick, have you ever taken your child to see the VHT?
a. Yes
b. No (GO TO QUESTION 328)
c. No response (GO TO QUESTION 328)

READ: Thinking of the last time you took a sick child under 5 years old to see the VHT.

315 When you decided to take your child to see the VHT, was the VHT available promptly?
a. Yes
b. No
c. No response

316 Were you able to see the VHT within 4 hours of deciding your child needed to be seen?
a. Yes
b. No
c. No response

317 How would you rate experience of being **treated with respect** by the VHT?
a. Unacceptable
b. Not very good
c. Acceptable
d. Above average
e. Excellent
f. No response

318 How was your experience of **communication** with the VHT while he/she was caring for your child?
a. Unacceptable
b. Not very good
c. Acceptable
d. Above average
e. Excellent
f. No response

319 How would rate this experience of the VHT caring for your child?
   a. Poor
   b. Fair
   c. Good
   d. Very good
   e. Excellent
   f. No response

320 Was your child was referred to a health centre by a VHT for further treatment?
   a. Yes
   b. No (GO TO QUESTION 324)
   c. No response (GO TO QUESTION 324)

321 Did you take the child to the health centre?
   a. Yes
   b. No (GO TO QUESTION 323)
   c. No response (GO TO QUESTION 324)

322 When did you go to the health centre?
   a. Within 1 day of referral
   b. Within 2-3 days of referral
   c. Within 1 week
   d. After more than 1 week
   e. Never
   f. No response

(GO TO QUESTION 324)

323 Why did you not go to the health centre?
   a. Lack of money
   b. Lack of time
   c. Lack of trust in provider
   d. Lack of transport
   e. Lack of approval by spouse
   f. Health center without medications
   g. Other (specify):____
   h. No response

324 In the past month, has any child under 5 years old in this household been assessed by a VHT?
   a. Yes
   b. No (GO TO QUESTION 328)
   c. No response (GO TO QUESTION 328)

325 If yes, how many times have in the past month has a child under 5 years old been assessed by a VHT? _______________

326 In the past month, how many times did a VHT assess any child under 5 years old in your household and refer the child on to the health centre?

____

327 If your child was referred to a health centre by a VHT for treatment, did you take the child to the health centre?

a. Every time
b. Most of the times
c. Sometimes
d. Never
e. No response

328 How many times in the past month has a member of your household visited a health center for a child under 5 years old? (enter 99 if does not know)

____

329 If your child is sick and you think they need treatment, where do you usually go first?

a. Health centre
b. VHT
c. Traditional medical practitioner
d. Drug Shop
e. Other (specify): ____
f. No response

330 How often do you contact your VHT if a child is sick?

a. Rarely
b. Sometimes
c. Most of the time
d. Every time
e. No response

331 How do you usually contact your VHT if a child is sick?

a. Phone/SMS
b. Go to VHT's house
c. Send someone to get VHT to bring to your household
d. Other:____
e. No response

332 Have you ever had a sick child and been unable to find a VHT?

a. Yes
b. No
c. No response

333 How would you rate your overall experience with VHTs in the past?

a. Unacceptable
b. Not very good
c. Acceptable
d. Above average

e. Excellent
f. No response

334 How confident are you in the abilities of the VHT?
a. Not confident
b. Somewhat confident
c. Confident
d. Very confident
e. No response

335 How confident are you in the abilities of the VHT to manage **cough**?
a. Not confident
b. Somewhat confident
c. Confident
d. Very confident
e. No response

336 How confident are you in the abilities of the VHT to manage **diarrhea**?
a. Not confident
b. Somewhat confident
c. Confident
d. Very confident
e. No response

337 How confident are you in the abilities of the VHT in the manage **fever**?
a. Not confident
b. Somewhat confident
c. Confident
d. Very confident
e. No response

338 Would you have your child seen by a VHT in the future if he or she had a health problem?
a. Yes
b. No
c. No response

D. MALARIA, PNEUMONIA, AND DIARRHEA IN THE COMMUNITY

READ: Now I would like to ask you some questions about malaria, pneumonia, and diarrhea in the community.

401 How serious a problem is pneumonia in this community?

a. Not a problem
b. A minor problem
c. A major problem
d. The most important health problem
e. No response

402 How serious a problem is diarrhea in this community?

a. Not a problem
b. A minor problem
c. A major problem
d. The most important health problem
e. No response

403 How serious a problem is malaria in this community?

a. Not a problem
b. A minor problem
c. A major problem
d. The most important health problem
e. No response

404 Do VHTS in your community currently have medicines available to treat children who are sick?

a. Yes
b. No
c. No response

405 Does your household have any mosquito nets that can be used while sleeping?

a. Yes
b. No (GO TO QUESTION 419)
c. Don't know (GO TO QUESTION 419)

406 How many mosquito nets does your household have? _______

(Ask each question for each net, going through each question in order for the first net, then the second and third, until you have discussed all nets. Make sure to complete all the numbered questions – numbers 407 - 418).

Question	Net 1	Net 2	Net 3
May I have a look at each of your mosquito nets?	407. Net 1 observed?	411. Net 2 observed?	415. Net 3 observed?

	**1 Yes / 2 No**	**1 Yes / 2 No**	**1 Yes / 2 No**
(If you're able to observe the net, the net in good or poor condition?<br><br>If you are not able to observe the net, ask the respondent: "Is the net in good or poor condition?"	408. Net 1 holes?<br><br>**1. Good**<br>**2. Poor**<br>**3. Don't know**	412. Net 2 holes?<br><br>**1. Good**<br>**2. Poor**<br>**3. Don't know**	416. Net 3 holes?<br><br>**1. Good**<br>**2. Poor**<br>**3. Don't know**
Did you purchase this mosquito net or were you given it for free (without cost)?	409. Net 1<br><br>**1. Purchased**<br>**2. Given for free**	413. Net 2<br><br>**1. Purchased**<br>**2. Given for free**	417. Net 3<br><br>**1. Purchased**<br>**2. Given for free**
How many children under 5 years slept under this net last night?	410. Net 1 children under 5: _______	414. Net 2 children under 5: _______	418. Net 3 children under 5: _______

(If there are more than 3 nets, use the additional net sheet)

419 In your opinion, what causes malaria? (Do not read list; circle ALL answers given)

a. Mosquito bites
b. Eating matooke
c. Eating maize
d. Eating mangoes
e. Eating dirty food
f. Drinking unboiled water
g. Getting soaked with rain
h. Cold/changing weather
i. Witchcraft
j. Contact with infected person
k. Other (specify) ____
l. No response

420 What is the main way that you learn about health problems such as malaria?

a. Family, friends, or other community members
b. Community meetings
c. VHT
d. Health center and staff
e. Radio/television
f. Newspaper or magazine
g. Other (specify):____
h. No response

E. WOMEN'S HEALTH

READ: Now I would like to ask you some questions about women's health and child bearing. We want to re-iterate that all responses are completely private and that you do not need to answer any question that with which you are uncomfortable.

501 Is it okay to proceed with questions about this area?
  a. Yes
  b. No (GO TO QUESTION 601)

502 How many pregnant women are currently in your household? __________________

(If "0", GO TO QUESTION 504)

503 How many pregnant women slept under a mosquito net last night? __________________

{If respondent is NOT a woman, please go to question 529}

504 Are you pregnant currently?
  a. Yes
  b. No (GO TO QUESTION 512)
  c. No response (GO TO QUESTION 512)

505 When was you last menstrual period? __/__/___ (DD/MM/YYYY)

506 How many antenatal care appointments have you attended for this pregnancy? _________

(If "0" antenatal care appointments, go to question 509)

507 Where did you go for antenatal care? (CIRCLE ALL THAT APPLY)
  a. Health centre
  b. Traditional birth attendant
  c. Drug shop
  d. Other: ____
  e. No response

508 Who attended the antenatal care with you? (CIRCLE ALL THAT APPLY)
  a. Spouse
  b. Mother
  c. Mother-in-law
  d. Other family: ____
  e. Friend
  f. No one else, attended alone
  g. No response

509 Are you planning on attend any further antenatal care at a health centre?
  a. Yes
  b. No

c. No response

510 Where do you plan to give birth?
a. Health centre 2 (GO TO QUESTION 512)
b. Health centre 3 (GO TO QUESTION 512)
c. Health centre 4 (GO TO QUESTION 512)
d. District hospital (GO TO QUESTION 512)
e. Private hospital/clinic (GO TO QUESTION 512)
f. Your Home
g. Another's home
h. No response (GO TO QUESTION 512)

511 Who will assist you to deliver the baby in the home?
a. Traditional birth attendant
b. Family member
c. Health center staff
d. No one
e. Other: _____________
f. No response

512 Have you ever been pregnant before in the past?
a. Yes
b. No (GO TO QUESTION 524)
c. No response (GO TO QUESTION 524)

513 How many live births have you had? ____________

514 What was your age at the time of your first pregnancy? ___________

515 How many times have you been pregnant previously? ______________

516 In the past where have you given birth: (CIRCLE ALL THAT APPLY)
a. Health centre 2
b. Health centre 3
c. Health centre 4
d. District hospital
e. Private hospital/clinic
f. Your Home
g. Another's home
h. No response

517 With the most recent delivery, where did you give birth:
a. Health centre 2
b. Health centre 3
c. Health centre 4
d. District hospital
e. Private hospital/clinic
f. Your Home

g. Another's home
h. No response

518 If not at your home, who accompanied you to the site of the delivery?
a. Spouse
b. Mother
c. Mother-in-law
d. Other family: ____
e. Friend
f. No one else, attended alone
g. Delivered at my home
h. No response

519 Who assisted you to deliver the baby in the home?
a. Traditional birth attendant
b. Family member
c. Health center staff
d. No one
e. Other: ____________
f. No response

520 If you did not deliver at a health facility, why did you not go to the health facility?
a. Lack of time
b. Lack of money
c. Personal preference (you rather be at home)
d. Other family members' preference including spouse, mother-in-law, or mother
e. Other: ______________________________
f. No response

521 If you delivered at a health facility, was the delivery:
a. Vaginal delivery
b. C-section

522 Did any health worker visit you after delivery?
a. VHT
b. Traditional birth attendant
c. Health center staff member
d. Other: ______________________
e. No response

523 How many living children do you have? ______________

524 How many children do you desire to have in your whole life? ___________

525 How many children do you think your current partner desires to have in his life? ___________

526 If you desire to have more children, would you want to have another child in the next 12 months?

a. Yes
b. No
c. Undecided
d. No response

527 Have any women from this household died during child birth in the last year (12 months)?

a. Yes
b. No
c. No response

528 What is the main way that you learn about women's health including pregnancy, delivery, and contraception?

a. Family, friends, or other community members
b. Traditional birth attendants
c. Health center and staff
d. Community meetings
e. VHT
f. Health center and staff
g. Radio/television
h. Newspaper or magazine
i. Other (specify):____
j. No response

(PLEASE GO TO QUESTION 601)

FOR MEN

529 Is your partner currently pregnant?

a. Yes
b. No (GO TO QUESTION 532)
c. No response (GO TO QUESTION 532)

530 During this pregnancy, has she attended antenatal care?

a. Yes
b. No
c. Do not know
d. No response

531 During this pregnancy, have you attended any antenatal care appointments with her?

a. Yes
b. No
c. Do not know
d. No response

532 How many times has your partner been pregnant previously? _______________

(If "0", GO TO QUESTION 539)

533 In the past, when pregnant, did she attend antenatal care?
   a. Yes
   b. No
   c. Do not know
   d. No response

534 Did you attend any antenatal care appointments with her?
   a. Yes
   b. No
   c. No response

535 In the past where has your partner given birth? (Circle all that apply)
   a. Health centre 2
   b. Health centre 3
   c. Health centre 4
   d. District hospital
   e. Private hospital/clinic
   f. Your Home
   g. Another's home
   h. Do not know
   i. No response

536 With the most recent delivery, where did your partner give birth:
   a. Health centre 2
   b. Health centre 3
   c. Health centre 4
   d. District hospital
   e. Private hospital/clinic
   f. Your Home
   g. Another's home

537 If delivered at home, who assisted her to deliver the baby in the home?
   a. Traditional birth attendant
   b. Family member
   c. Health center staff
   d. No one
   e. Other: _____________
   f. No response

538 If she did not deliver at a health facility, why did she not go to the health facility?
   a. Lack of time
   b. Lack of money
   c. Personal preference (you rather be at home)
   d. Other family members' preference including spouse, mother-in-law, or mother
   e. Other: _____________
   f. No response

539 How many children do you want to have in your whole life? ___________

540 How many children do you think your current partner desires to have in her life? ___________

541 If you desire to have more children, would you want to have another child in the next 12 months?
   a. Yes
   b. No
   c. No response

542 Have any women from this household died during child birth in the last year (12 months)?
   a. Yes
   b. No
   c. No response

F. NONCOMMUNICABLE DISEASES

READ: Now, in this last section, I am going to ask you a few questions about some chronic diseases such as heart disease and risk factors for their development.

601 Do you currently smoke or use any tobacco products such as cigarettes, pipes, or chewing tobacco?
- a. Yes
- b. No (GO TO QUESTION 604)
- c. No response (GO TO QUESTION 604)

602 In the past 12 months, what types of tobacco have you used? (Circle all that apply)
- a. Cigarettes
- b. Pipes
- c. Smokeless tobacco (ie. Chewing tobacco)
- d. No response

603 In the past 12 months, how frequently have you used tobacco?
- a. Daily
- b. 5-6 days per week
- c. 1-4 days per week
- d. 1-3 days per month
- e. Less than once a month
- f. No response

604 Does anyone else in your household use any tobacco products such as cigarettes, pipes, or chewing tobacco?
- a. Yes
- b. No
- c. No response

605 Have you consumed alcohol (beer, wine, spirits) in the last 12 months?
- a. Yes
- b. No (GO TO QUESTION 607)
- c. No response

606 In the past 12 months, how frequently have you had at least 1 drink of alcohol?
- a. Daily
- b. 5-6 days per week
- c. 1-4 days per week
- d. 1-3 days per month
- e. Less than once a month

607 How many people in your household have been diagnosed with the following conditions:
- a. Hypertension? ____________
- b. Heart disease? ____________
- c. Diabetes? ____________
- d. Asthma? ____________

e. Stroke? ____________
f. Depression? ____________
g. Seizures? ____________

THANK YOU FOR PARTICIPATING IN OUR SURVEY!

F) Additional net sheet

Question	Net 1	Net 2	Net 3
May I have a look at each of your mosquito nets?	450. Net 1 observed?<br><br>**1 Yes / 2 No**	454. Net 2 observed?<br><br>**1 Yes / 2 No**	458. Net 3 observed?<br><br>**1 Yes / 2 No**
(If you're able to observe the net, the net in good or poor condition?<br><br>If you are not able to observe the net, ask the respondent: "Is the net in good or poor condition?"	451. Net 1 holes?<br><br>**1. Good**<br>**2. Poor**<br>**3. Don't know**	455. Net 2 holes?<br><br>**1. Good**<br>**2. Poor**<br>**3. Don't know**	459. Net 3 holes?<br><br>**1. Good**<br>**2. Poor**<br>**3. Don't know**
Did you purchase this mosquito net or were you given it for free (without cost)?	452. Net 1<br><br>**1. Purchased**<br>**2. Given for free**	456. Net 2<br><br>**1. Purchased**<br>**2. Given for free**	460. Net 3<br><br>**1. Purchased**<br>**2. Given for free**
How many children under 5 years slept under this net last night?	453. Net 1 children under 5: ______	457. Net 2 children under 5: ______	461. Net 3 children under 5: ______
